# Underwater Breathing
*Received October, 1952.


**Published:** 1953

**Authors:** Ronald Woolmer

**Affiliations:** Surgeon Commander, R.N.V.R.


### UNDERWATER BREATHING*

BY

RONALD WOOLMER, F.F.A.R.C.S.

*Surgeon Commander, R.N.V.R.*

The pressure of the atmosphere at sea-level is commonly disregarded, because e are perfectly adapted to it, but we must know something about it if we are to nderstand the effect of departures from it.

The weight of the atmosphere at sea-level exerts a pressure of 15 lb. p.s.i., and will balance a column of sea-water 33 ft. high, or a column of mercury 30 in. '60 mm.) high. As long as the glottis is open, this pressure is exerted equally n the inside and outside of the chest.

A diver in a non-rigid suit 33 ft. below the surface is exposed to 2 atmospheres ressure, or 30 lb. p.s.i. This is the pressure on the outside of his body, and if is chest is not to collapse the air inside it must be at the same pressure.

At 300 ft. the pressure is 10 atmospheres, and the air supplied to the diver ıust be at this pressure too. The partial pressure of $O_2$ to which the pulmonary lood is exposed at 10 atmospheres increases more than tenfold, because the artial pressures of water and $CO_2$ do not rise, and it approaches 1500 mm.

This pressure pushes so much oxygen into solution in the plasma that the ssues have to draw little or none of the $O_2$ carried in the haemoglobin. This onsequently remains unreduced. This in turn means that there is less base vailable to combine with $CO_2$, and the carriage of $CO_2$ away from the tissues ıay be seriously interfered with.

Similarly nitrogen is driven into solution in the plasma and tissue fluids in reat quantities. So long as the pressure driving it in is maintained this does little hysical harm, though it may produce nitrogen narcosis. This is an impairment of erebral function, similar to alcoholic intoxication, which is frequently experi- ıced by divers. Other inert gases have a similar effect, in proportion to their ıolecular weights. Xenon has recently been shown to have narcotic properties, ıd argon, which in air at 10 atmospheres exerts a pressure of about 70 mm. Hg., lso contributes to the narcosis. This problem of nitrogen narcosis is a very real ne. There is a great variation in susceptibility, but many divers are more or ss incapacitated at depths of about 300 ft., where the partial pressure of nitrogen 6000 mm. of mercury.

The nitrogen is, however, an even greater nuisance during ascent. Tissues ary in the amount of nitrogen they can hold in solution at a given pressure, and ı the speed with which they take up that amount. The curve of uptake plotted gainst time is exponential, so we talk about half saturation times, and the slower ıturating tissues (such as ligaments and cartilage) take about 75 min. to absorb alf their load. Similarly, if the pressure is rapidly reduced after complete

* Received October, 1952.

saturation has been achieved, it will be 75 min. before they have given of[illegible] their excess nitrogen, and several hours before they have parted with it al[illegible]

During decompression, the excess nitrogen forms bubbles in the tis[illegible] which may give rise to "bends", by which we mean pain in or near a joint, [illegible] bubbles in the bloodstream.

Not only is the speed of ascent limited by this factor, but also the leng[illegible] time spent at working depth, because the more nearly the " slower " tis[illegible] reach saturation the greater will be the amount of excess nitrogen to be got ri[illegible]

Haldane investigated this problem in 1908. He found that body tis[illegible] differed from water in being able to become " supersaturated " with nitro[illegible] They can hold nitrogen at about twice the tension of the ambient atmosp[illegible] without forming bubbles of it; and provided the tension in the ambient at[illegible] sphere is not less than half that in the tissues bubbles will not be formed[illegible] matter how quickly the pressure drops. Haldane reasoned that if the total p[illegible] sure was halved the volume of nitrogen liberated would be the same wh[illegible] the reduction was from 10 atmospheres to 5, or from 2 to 1. It followed[illegible] decompression by stages, halving the total pressure at each stage, would en[illegible] the diver to get rid of excess nitrogen far more quickly than if he came up [illegible] even rate.

This led to the system of Stage Decompression—based on Haldane's Dec[illegible] pression Tables—which was a great advance on the older system, and which [illegible] adopted throughout the world.

Experience in more recent times, however, has shown that the theory doe[illegible] quite fit the facts, just as Newton's theory of gravity did not quite fit the [illegible] which improved measuring instruments were able to reveal.

It was found, for instance, that Haldane's Safe Decompression Ratio of 2 wa[illegible] low for deep dives and too high for shallow ones, and his tables had to be alt[illegible]

(The Safe Decompression Ratio is the highest ratio of nitrogen tension in the body to that outside it at which bubbles do not form.)

Very recently Hempleman, working at the R.N. Physiological Laborato[illegible] Portsmouth, has made a new approach to the problem with brilliant results[illegible]

Briefly, he has shown that the amount of excess nitrogen in the tissues v[illegible] as $D\sqrt{t}$, where $D$ is the depth of the dive in feet and $t$ the time of exposu[illegible] minutes. If this value, in " nitrogen units ", is kept below 500 for the goat[illegible] bends will occur on rapid decompression. Goats are rather more susceptib[illegible] bends than humans, but to give an adequate safety margin the figure for hum[illegible] has been set at 475. Hence a diver can remain at 33 feet for nearly 4 hours [illegible] come up without a stop. ($33\sqrt{t} = 475$, $t = 225$ min.); but if he descend[illegible] 100 ft. his time below, if he is to come up without a stop, is limited to 22½ [illegible] ($100\sqrt{t} = 475$; $t = 22{\cdot}5$). If he has to stay down for longer than this, [illegible] decompression will have to be used. The depth and duration of each sto[illegible] the way up can be calculated from the formula.

New tables based on this simple theory have been worked out, and are [illegible] being tested by volunteers in the compression chamber at the Royal N[illegible] Physiological Laboratory in a carefully controlled series of experiments. [illegible] results so far are most promising, and there is little doubt that the new sch[illegible] will replace Haldane's and will result in better performances.

The threat of bends imposes unwelcome limitations on the diver, and [illegible]

ey are caused by nitrogen bubbles it would seem that they might be avoided y using another gas than air. If the diver is supplied with compressed oxygen nstead of compressed air, bubbles form much less readily and the danger of ends is almost eliminated. It is replaced, however, by a greater danger; that of xygen poisoning. Oxygen exerting a partial pressure of over 3 atmospheres, hich would be attained by breathing the pure gas at 66 ft., or air at over 500 ft., poisonous. It causes twitching, convulsions, unconsciousness and death, ometimes after quite short exposures.

The nitrogen in air cannot therefore be replaced by oxygen at depths greater han 60 ft., and indeed the oxygen content of ordinary air is too great to permit s use in very deep diving, and a mixture less rich in oxygen has to be used for his. However, oxygen is used to replace air when the diver has ascended to a vel where it will not be poisonous. This hastens the elimination of nitrogen, nd enables the remainder of the ascent to be completed much more quickly.

The replacement of nitrogen by helium confers certain advantages. Helium less soluble and more diffusible than nitrogen, so that the tissues attain equilirium with it more quickly. Similarly, it leaves the tissues faster during decomression. The safe decompression ration is less for helium (about 1·7) than for itrogen (about 2), and in stage decompression with helium the first stop has herefore to be deeper than with nitrogen. However, helium under increased ressure causes no intoxication, presumably because of its low molecular weight, nd herein lies its great advantage over oxygen. It has permitted much greater epths to be attained, and it was on a helium-oxygen mixture that P.O. Bollard ith H.M.S. *Reclaim* at Loch Fyne reached the depth of 540 ft. (17½ ats. abs.) 1948. This depth has not yet been surpassed.

An interesting point about helium is that it is a much better conductor of heat han is nitrogen, and divers using an oxygen-helium mixture have to be provided ith electrically-warmed suits to prevent them from getting chilled.

For shallower diving, mixtures of nitrogen and oxygen have been developed t the Admiralty Experimental Diving Unit. These were required in particular r the Mine Recovery Units, which had to be quickly developed for clearing arbours after D-Day. For this work self-contained suits were required, as it as not considered justifiable to risk blowing up more than one man. A lone iver cannot use stage decompression to avoid the bends, so mixtures rich in itrogen could not be safely used. Similarly, a lone diver dare not risk oxygen oisoning, so he may not use pure oxygen to avoid nitrogen bubbles. A mixture f nitrogen and oxygen, in suitable proportions, though not guaranteeing the voidance of either, will considerably reduce the risk of both, and the self-conained Mine Recovery Suit, using such a mixture, has been very successful. The act that all this equipment, down to the last nut and bolt, had to be nonagnetic greatly complicated its manufacture.

The submariner and the frogman have raised many interesting problems in espiratory physiology, and much good work has been done in back rooms to olve them.

A submarine usually carries a complement of about sixty men. It may have o remain submerged for long periods, and the atmosphere must remain respirble. Submarines carry very efficient soda lime canisters for absorbing carbon ioxide, chemical oxygen generators and oxygen compressed in cylinders.

The trend is always towards making the submarine a real undersea boat—o that will remain submerged for a month on end—and this raises difficult stora problems. In a month a submarine's complement would consume nine tons $O_2$ and produce eight tons of $CO_2$, so the magnitude of the problem is appare It would be much easier to prevent the $CO_2$ from rising above a certain level—s 2 per cent.—than to keep it right down to physiological limits. What is the ma mum level of $CO_2$ that can be borne without ill effects for a month? Nobo knows. All previous work has been in the nature of short-term experime There is some evidence to show that a few hours' exposure to 2 per cent. $CO_2$ w result in severe symptoms if the $O_2$ is at the same time reduced to about 16 p cent. of one atmosphere, but that it causes no symptoms if the $O_2$ tension normal. A large-scale experiment, in which a fully-manned submarine will shut down for a month, is planned to settle this question.

The " snort " or breathing tube introduced to submarines during rece years also brings physiological problems with it. A submarine's diesel engin take in air at a tremendous rate. With the hatches closed, and the ship runni below the surface, all this air has to come through the snort tube. But the sno leaves a tell-tale wake, so it has to be kept small, and the required flow of air c only just get through it. Hence there is quite a steep pressure gradient along t tube, and during snorting the air in the submarine is below atmospheric pressu The pressure is often as low as 600 mm., equivalent to an altitude of nea 7,000 ft. which would give a partial pressure of oxygen of only 80 mm. in t alveolar air. But if the sea is choppy conditions are more interesting. If a wav let tries to get into the top of the snort tube, which may be less than a foot abo the surface, the float valve automatically closes, and remains closed until the wa has gone by. But the diesels don't stop devouring air, and the pressure fa steeply until the valve reopens, when it regains its previous level. This corre ponds to being repeatedly shot up to about 15,000 ft. altitude and down again 6,000 ft., and the effect can be quite disconcerting, especially on the eardru When it becomes possible to do away with internal combustion engines whic require atmospheric air, the snort and its attendant problems will disappear.

The most interesting physiological problems, however, are associated wit escape from sunken submarines. There are four possible methods. The pressu hull of a submarine, housing the crew, is a rigid tube in which the air is kept atmospheric pressure. The depth to which a submarine can go is limited by t strength of this tube. At depths greater than a few hundred feet, the hull wou be crushed by the pressure of sea-water, and if a submarine is holed and sin to crushing depth there is no question of escape. Practical methods are therefo concerned only with escapes from up to three or four hundred feet. Three o the four methods involve compression of the crew to the ambient pressure, wit subsequent decompression during ascent. In the fourth method, the diving be in which a second rigid tube is connected to the pressure hull, the crew rema at atmospheric pressure all the time.

The first method is compartment escape. The pressure hull of a submari is divided into four or five watertight compartments, each with its escape hatc After an accident, survivors are collected into one of these compartments (or mor if more than one remains intact). The escape hatch in the top of the compartme is surrounded by a collapsible trunk of rubberized fabric, which can be draw down as a vertical tube down to within a few feet of the deck, and rigidly secured. The sea cocks are opened, and water floods into the compartment, compressing the air above it as it rises. If the vessel is lying at a depth of 130 ft., where the pressure is 5 atmospheres, the air in the compartment will be compressed to one-fifth of its volume, and when the water has reached four-fifths of the distance to the top of the compartment, no more will come in. The crew have their heads above water, and they are breathing compressed air. The first man then puts on his under-water breathing set, dives under the rim of the trunk, and comes up inside it. Since the pressures on each side of the escape hatch are now equal, it can be opened, and the man opens it and floats to the surface. The others follow, one by one, at intervals of 15–30 seconds. Flooding the chamber need not take more than a few minutes, and during that time the pressure in the compartment increases from 1 to 5 atmospheres. The ears have to be cleared repeatedly as the pressure comes on.

Sensations during flooding up are peculiar, particularly if the depth is over 100 ft. One feels the water squeezing the legs and body with a vicelike grip, and the clothes are pressed flat and wrinkled against the surface of the body. The eardrums bulge, and quickly become painful if they are not promptly cleared. It soon becomes impossible to whistle, owing to the increased density of the air, and the air current caused by moving the hand close to the head will blow the hair about. No difficulty is felt in moving the heavy air in and out of the lungs when the body is at rest; but it becomes an effort with exercise. The voice has a peculiar bleating quality, and this often causes much hilarity—at any rate in practice dives—among the mildly intoxicated personnel. This intoxication is due, of course, not to an issue of rum, but to high partial pressures of nitrogen. In wartime escapes, however, there is not much hilarity during flooding up, as the water's icy grip climbs up the body. The lights have probably failed, and the air may be already foul after prolonged submergence, and perhaps contaminated with chlorine or smoke. Some of the crew will probably have been drowned, and all shaken by depth-charging. They know that if they get to the surface they may find themselves in an extremely unfriendly environment.

The man in command of the group, after an accident, has to decide when to order the compartment escape to begin, and the decision may be a difficult one. He may wish to delay until surface forces have located the submarine, or until daylight and slack tides. The time he can afford to wait will depend upon the number in the compartment, its size, and how long the submarine had been submerged before the accident. It will also depend on whether the air-purifying system is still workable, and on how many of the survivors have submarine escape sets. Rising $CO_2$ and falling $O_2$ levels may force his hand. Once he has decided on escape and the chamber has been flooded, the men waiting to go up are exposed to new dangers. Those without breathing sets are building up their tissue nitrogen and have the bends in store, and their cerebration and survival is imperilled by nitrogen narcosis and $CO_2$ poisoning. Two per cent. $CO_2$—quite a usual level in a submerged submarine—will exert the same tension, after flooding up at 130 ft., as 10 per cent. would at normal pressure. At the tension of oxygen which will go with it, it will produce stupidity and unconsciousness rather than respiratory stimulation. Those with breathing sets may avoid the bends, nitrogen narcosis and $CO_2$ poisoning, but they are faced with oxygen poisoning, and in any case the duration of a set is only about forty-five min[illegible] It is therefore imperative, once the chamber has been flooded, to get the me[illegible] as quickly as possible.

Compartment escape was the method used in H.M.S. *Truculent*, which [illegible] holed while on the surface in the Thames Estuary. There were seventy-[illegible] men aboard. Five, on the bridge, were swept into the water as she sank. [illegible] were drowned by the flooding of their compartments. The remaining sixty-[illegible] managed to close the watertight doors and secured themselves in two comp[illegible]ments. Every one of these sixty-four escaped alive from the submarine. [illegible] tragedy was that owing to a series of mischances only ten were picked up [illegible]

The two other methods of escape under pressure are tower escape, from [illegible] conning-tower or gun-tower hatch, and individual chamber escape, fr[illegible] chamber specially fitted in some submarines. In these methods a small cha[illegible] able to hold one or two men, is repeatedly occupied, flooded up, opened t[illegible] sea, evacuated, closed from the sea, drained, restored to atmospheric pres[illegible] opened to the interior, occupied and so on. It is a slow method, but the [illegible] waiting to escape remain dry and at normal pressure, which may be an advan[illegible] It was by individual chamber escape that the few survivors of H.M.S. *Thetis* go[illegible]

In the diving-bell method the bell is lowered over the escape hatch of the [illegible] marine and fastened on. The pressures are then equalized, the hatch op[illegible] and two, three or four people pass into the bell. The doors are closed, the [illegible] broken, and the bell raised to the surface. This process has to be repeated [illegible] times. The method is comfortable, in that the survivors remain dry and at [illegible] mal pressure, but it is slow and can only be applied in favourable circumsta[illegible] It was used very successfully with the American submarine *Squalus* in 19[illegible]

An underwater breathing set, though useful, is not essential to escape, an[illegible] method of " Free Ascent " is less hazardous than it sounds. It is taught [illegible] practised regularly from up to 100 ft., and free ascents have been made from [illegible] and even 300 ft.

At 100 ft. the air in an escape compartment which has been flooded up [illegible] be at 4 atmospheres pressure. The partial pressure of oxygen will therefore [illegible] one-fifth of 4 atmospheres, and the effect will be equivalent to breathing [illegible] cent. oxygen at sea-level. It is well known that after deep breathing of oxy[illegible] rich mixtures it is possible to hold the breath for a long time. Rates of [illegible] in sea-water vary from 1 to 4 ft. a second, so an ascent from 100 ft. will [illegible] take less than a minute. There is no difficulty in *holding* one's breath fo[illegible] time. The difficulty is to get rid of it.

Suppose the lungs contain 5 litres of air at the start of the ascent from [illegible] At 66 ft. (3 atmospheres) this air will have expanded to 6·7 litres, at 33 [illegible] 10 litres, and at the surface to 20 litres. If it is not allowed to escape fre[illegible] will burst the lungs, and the volume to be got rid of per second increases [illegible] ithmically during the ascent. In the first third of the journey 1·7 litres mu[illegible] vented: in the second 2·5, and in the last third 5. Even if the pressure do[illegible] rise quickly enough to burst the lungs, it may break through the suppo[illegible] tissues at the periphery. This may result in interstitial emphysema or m[illegible] air embolism after surfacing.

Obeying the natural instinct to hold the breath while under water may [illegible] fore have fatal results. This instinct can be conquered by training, and [illegible]

Thousands of successful free ascents have been made as part of the submariner's normal training. In the U.S. the 100-ft. tank has been in operation for several years. In Britain a 100-ft. tank has recently come into use, and smaller tanks have been working for many years.

These 100-ft. training tanks have airlocks at various levels. An airlock is a blister in the tank wall, big enough to accommodate a man. The lower part is filled with water, the upper two feet or so with air at the same pressure. At the bottom of the tank there is a chamber, similar to an escape compartment of a submarine, by which it is entered.

A number of instructors are stationed in the tank at various depths. These instructors remain below the surface for half an hour or so at a time. When an instructor needs air, he goes into one of the locks, puts his head above water, and breathes compressed air. A few deep breaths of this will last him for several minutes. Instructors become adept at moving about in the water with very little effort. They develop remarkable powers of manoeuvre, and become almost aquatic animals.

The pupil about to make a training ascent goes, with an instructor, into the airlock at the bottom. The outer door is closed and water is admitted, compressing the air in the lock. It rises above the top of the inner door, which is about four feet high. When the pressures are equal the inner door is opened. The pupil takes a few deep breaths, ducks under the water and out through the door into the main tank, and begins his ascent. The instructor for the bottom zone has meanwhile glided down from the 75-ft. lock and is waiting for the pupil as he emerges. He conducts him up through his zone, and hands him on to the next instructor. He then either returns to the bottom for the next pupil, or goes back to the 75-ft. lock for a breath of compressed air. When the organization is working smoothly an instructor may conduct two or three pupils through his zone before he needs to renew his air supply. The whole system is carefully co-ordinated, and controlled from the top by telephones and underwater loud-speakers.

A man who has made a free ascent from 100 ft. is pretty confident when it comes to escaping from a submarine. He knows that if artifice fails him he can depend on his own resources to rise uninjured to the surface.

The development of "human torpedoes", midget submarines, submersible canoes and "frogmen" and Mine Recovery Units during the war raised many interesting physiological problems, which had to be solved mainly by trial and error. These problems concerned not only underwater breathing, but also protection against extremes of temperature, the design of life-belts and buoyant suits that sustain an unconscious man, problems of underwater vision and illumination, protection against underwater explosions, barotrauma, being intelligible on an underwater telephone while wearing a breathing tube and mouthpiece, the best design for "frogmen's" feet, and so on; and they taxed the ingenuity of many medical officers, physiologists and engineers.

Work on all these problems is still going on.

### REFERENCES

Boycott, A. E., Demant, G. C. C. and Haldane, J. S.: *J. Hyg. Camb.*, 1908, **8**, 342. The prevention of compressed-air illness.

Hempleman, H. V.: *Med. Research Council Rep.*, R.N.P., 52/708. A new theoretical basis for the calculation of decompression tables.

